# Hydrological time series prediction based on IWOA-ALSTM

**DOI:** 10.1038/s41598-024-58269-3

**Published:** 2024-04-05

**Authors:** Xuejie Zhang, Hao Cang, Nadia Nedjah, Feng Ye, Yanling Jin

**Affiliations:** 1 Key Laboratory of Water Big Data Technology of Ministry of Water Resources, Hohai University, Nanjing, 211100 China; 2 School of Computer and Information, Hohai University, Nanjing, 211100 China; 3 State University of Rio de Janeiro, Rio de Janeiro, Brazil; 4 China South-to-North Water Diversion Jianghan Water Network Construction and Development Co., Ltd, Wuhan, China

**Keywords:** Engineering, Mathematics and computing

## Abstract

The prediction of hydrological time series is of great significance for developing flood and drought prevention approaches and is an important component in research on smart water resources. The nonlinear characteristics of hydrological time series are important factors affecting the accuracy of predictions. To enhance the prediction of the nonlinear component in hydrological time series, we employed an improved whale optimisation algorithm (IWOA) to optimise an attention-based long short-term memory (ALSTM) network. The proposed model is termed IWOA-ALSTM. Specifically, we introduced an attention mechanism between two LSTM layers, enabling adaptive focus on distinct features within each time unit to gather information pertaining to a hydrological time series. Furthermore, given the critical impact of the model hyperparameter configuration on the prediction accuracy and operational efficiency, the proposed improved whale optimisation algorithm facilitates the discovery of optimal hyperparameters for the ALSTM model. In this work, we used nonlinear water level information obtained from Hankou station as experimental data. The results of this model were compared with those of genetic algorithms, particle swarm optimisation algorithms and whale optimisation algorithms. The experiments were conducted using five evaluation metrics, namely, the RMSE, MAE, NSE, SI and DR. The results show that the IWOA is effective at optimising the ALSTM and significantly improves the prediction accuracy of nonlinear hydrological time series.

## Introduction

Studying hydrological time series can improve the economic efficiency and help to optimise basin reservoir scheduling, protect people’s lives and improve property safety. However, hydrological time series exhibit strong uncertainty, stochasticity and nonlinearity due to the influence of various factors, such as rainfall, climate and human activities. Therefore, the accurate analysis of hydrological time series is very difficult. For analysis purposes, the prediction of hydrological time series is highly important for flood and drought prevention. The use of different methods to analyse hydrological time series data is the basis for accurate hydrological time series^1^prediction. There are many statistical analysis models used for hydrological time series prediction, such as the autoregressive moving average (ARMA)^2,3^ and autoregressive integrated moving average (ARIMA)^4–8^ models. Hydrological time series exhibit nonlinear relationships, which limits the effectiveness of ARIMA models. Another approach is to use neural network-based models^9–11^. Gunathilake^12^ used artificial neural networks to predict water flow in river basins in Sri Lanka based on rainfall. These networks achieved good results. Zhang^13^ proposed an RNN-based time series model for the intelligent prediction of future water levels in different reservoirs, and this model achieved higher accuracy than did an ANN model. As an improvement of an RNN, LSTM introduces a memory gate structure to solve the gradient explosion problem of RNNs and is widely used in hydrological data prediction. Pranolo^14^ compared LSTM with the BPNN algorithm. The experimental results showed that the LSTM method outperformed the BPNN in predicting precipitation. Shweta^15^applied LSTM to monthly monsoon rainfall data from the Haryana, Delhi and Chandigarh subdivisions. The experiments showed that better results were achieved for nonlinear rainfall data. Sahoo^16^ explored the suitability of LSTM-RNN over RNN for hydrological time series. The experimental results showed that the LSTM-RNN method can be used to model low-flow HTS at Basantapur station in the Mahanadi River Basin, India, and can achieve satisfactory performance over RNNs and the naïve Bayes method. Le^17^conducted experiments on daily observed flow data from 7 hydrology stations, and the results showed that four LSTM-based models performed better and maintained greater stability than did FFNN and CNN models. These studies demonstrate the favourable performance of LSTM in hydrological time series prediction. During the construction of an LSTM neural network, the model hyperparameters play a crucial role, as their values significantly impact the prediction results. However, in the context of hydrology forecasting with LSTM, the hyperparameters are typically manually set by human experts, which can severely limit the model’s prediction performance.

To address the aforementioned shortcomings of neural network-based models, an improved whale optimisation algorithm named ALSTM, an LSTM-based neural network augmented with attention mechanisms,is proposed in this study. Specifically, to address the nonlinear nature of hydrological time series, for which it is difficult to construct a simulated ensemble, an attention mechanism is introduced between two layers of the LSTM-based network, enabling the prediction of nonlinear components of time series. Furthermore, to mitigate the problem regarding the setting of model hyperparameters, which has a large impact on the prediction accuracy and operation efficiency, the initialisation population and parameter update methods in the whale optimisation algorithm are improved. In addition, dynamic optimisation is implemented to determine the hyperparameters of the ALSTM model, further improving the prediction accuracy of the model for the nonlinear components of hydrological data.

## Basic methods, LSTM concepts and models

**Figure 1:**
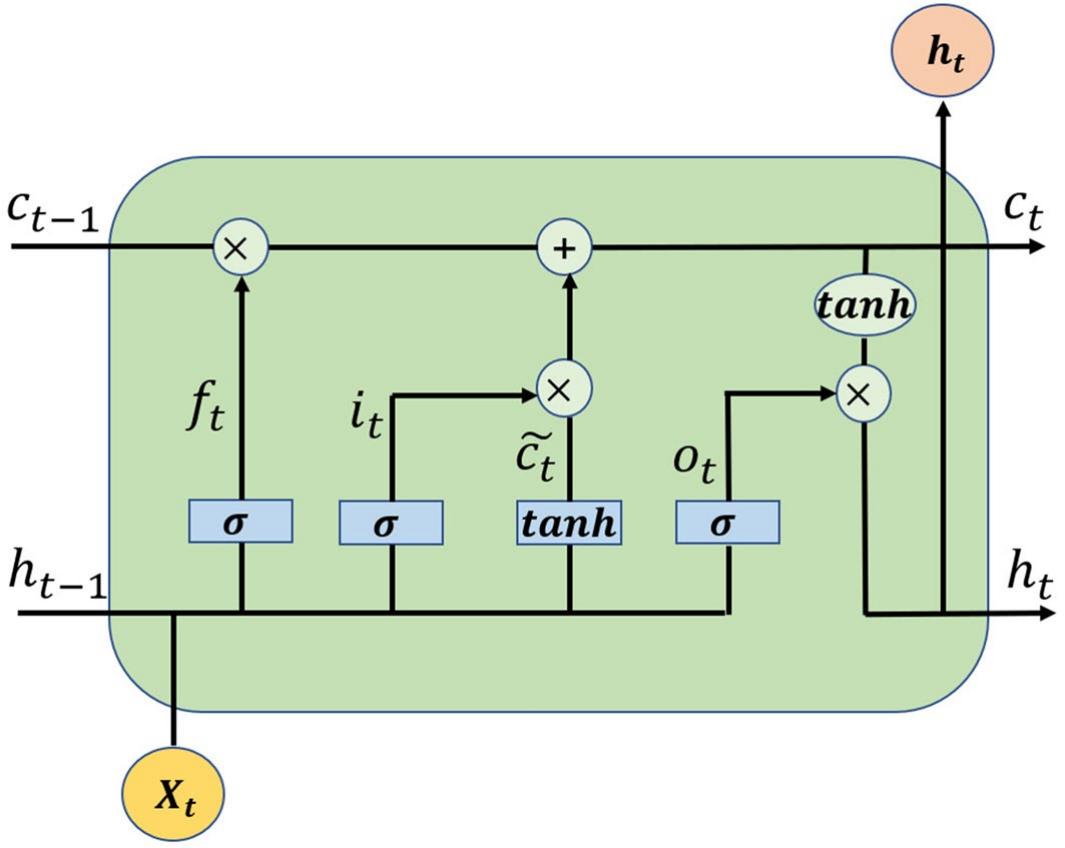
LSTM structure diagram.

LSTM is a special recurrent neural network (RNN)^18^ that solves the problems of gradient explosion and disappearance of RNNs in processing time series by introducing a gate mechanism to control which information is retained and forgotten^19^. LSTM is essentially a special kind of RNN. Compared with the conventional RNN, LSTM uses three cyclic gating units, an input gate, an output gate and a forgetting gate, to fully exploit the features of the temporal data. A schematic diagram of the LSTM structure of a single cell is shown in Fig. 1.

In the structure diagram, $h_{t-1}$ denotes the hidden state of the neuron at moment $t-1$, $C_{t-1}$ denotes the state of the memory unit of the neuron at moment $t-1$, $x_t$ denotes the input value at moment *t*, $f_t$, $i_t$, and $o_t$ denote the forget gate, the input gate, and the output gate, respectively, and $C_t$ denotes the status update value of the memory cell.

The forget gate $f_t$ in the LSTM determines which information the memory unit should discard. The forget gate reads the value $h_{t-1}$ of the previous hidden layer with the input value $x_t$ and subsequently outputs a vector between 0 and 1, where 0 indicates that all information in the information in the memory cell $C_{t-1}$ is forgotten and 1 indicates that all information is retained.

1 $$\begin{aligned} f_{t}=\sigma \left( W_{f} \cdot \left[ h_{t-1}, x_{t}\right] +b_{f}\right) \end{aligned}$$

The input gate $i_t$ determines whether new information is added to the memory cell. First, pass the input value $x_t$ and the information $h_{t-1}$ of the previous hidden layer are passed into the *sigmoid* activation function, and a vector $i_t$ is output with the same range of values as that of $f_t$.

2 $$\begin{aligned} i_{t}=\sigma \left( W_{i} \cdot \left[ h_{t-1}, x_{t}\right] +b_{i}\right) \end{aligned}$$

Then, using the input value $x_t$ and the information $h_{t-1}$ of the previous hidden layer, a new state value is output by the *tanh* activation function.

3 $$\begin{aligned} {\widetilde{C}}_t=tanh(W_C\cdot [h_{t-1},x_t]+b_C) \end{aligned}$$

Then, the memory cell $C_{t-1}$is updated. $C_{t-1}*f_t$ denotes the cell state of the previous layer multiplied by the forget gate to determine the information forgotten from $C_{t-1}$; $i_t*{\widetilde{C}}_t$ indicates that the sigmoid output value is multiplied by the *tanh* to determine the information added to the memory cell, and the two parts are weighted and summed to finally obtain the new information and update it into the cell state.

4 $$\begin{aligned} C_t=C_{t-1}*f_t+i_t*{\widetilde{C}}_t \end{aligned}$$

The output gate $o_t$ is used to determine the information for the next hidden layer input. First, the input value $x_t$ and the information $h_{t-1}$ of the previous hidden layer are fed into the sigmoid activation function.

5 $$\begin{aligned} o_t=\sigma (W_O\cdot [h_{t-1},x_t]+b_O) \end{aligned}$$

Then, the *tanh* activation function is applied to the updated memory cell information $C_t$, and finally the two activation function values are multiplied to obtain the state variable $h_t$ of the current hidden layer.

6 $$\begin{aligned} h_t=o_t*tanh(C_t) \end{aligned}$$

The internal structure of an LSTM network is more complex than that of a conventional RNN. The internal memory unit in LSTM is able to freely select the content of that memory in each time step, thus solving the problems of gradient explosion and gradient disappearance in RNNs and making LSTM more suitable for processing time series.

## Attention mechanism in LSTM

The long short-term memory(LSTM) neural network is adept at capturing long-term dependencies within hydrological time series data. The LSTM architecture incorporates gate units that enable the network to retain contextual memory from the hydrological time series, making it a widely employed technique for hydrological time series prediction. Nevertheless, LSTM processes sequential information incrementally during the prediction process, treating input data from each time step and feature equally. However, in practice, the proximity of the time intervals significantly influences the prediction outcomes. To address this issue, in this paper, an attention mechanism is introduced into the LSTM neural network. By placing the attention mechanism between two LSTM layers, the importance of various features at each time step can be assessed through attention weights, enabling the adaptive selection of input vectors with varying degrees of relevance and thereby enhancing the prediction accuracy.

**Figure 2:**
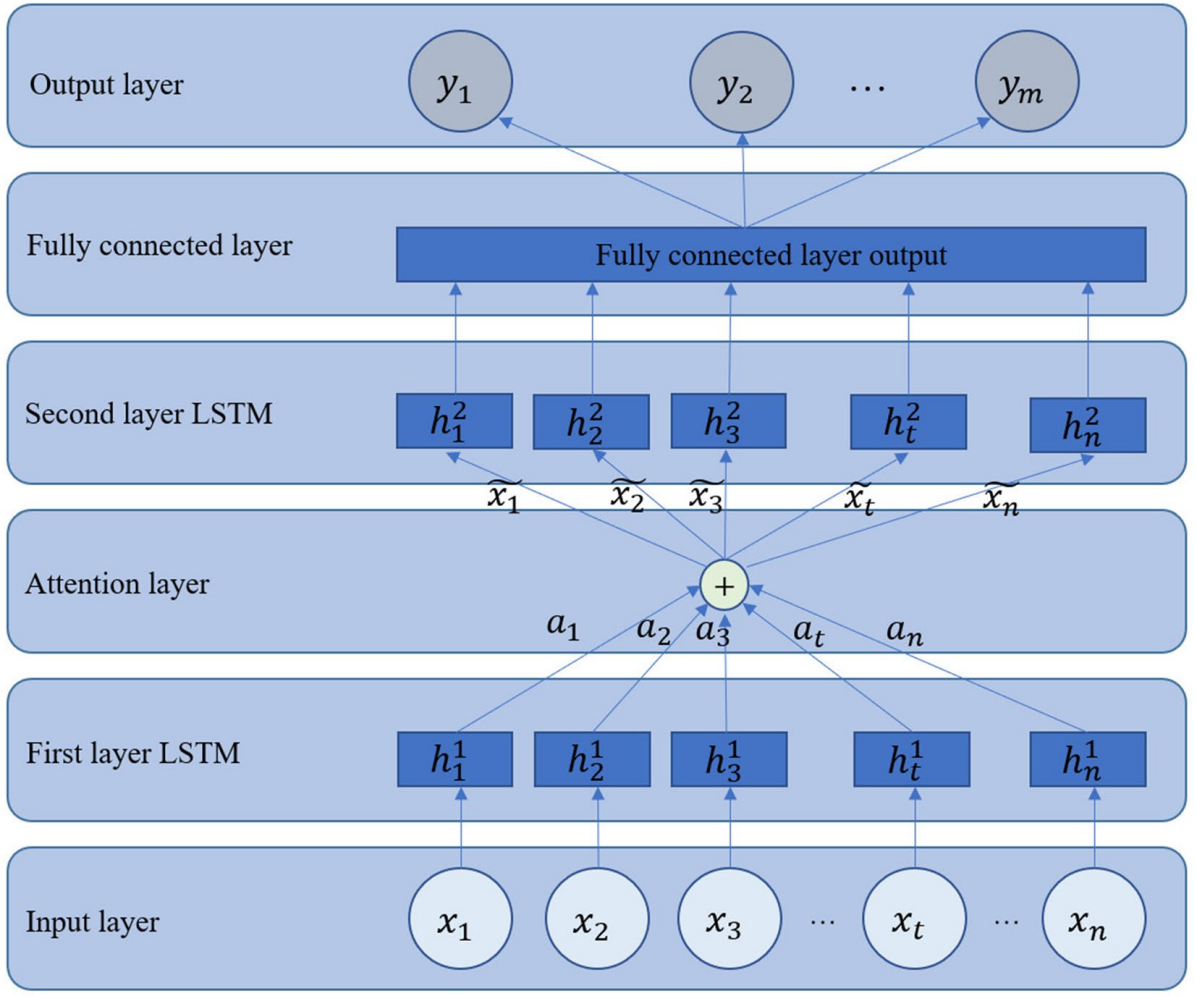
ALSTM network structure.

The ALSTM model consists of six parts: the input layer, the first LSTM layer, the attention layer, the second LSTM layer, the fully connected layer and the output layer. The ALSTM hydrological prediction model is shown in Fig. 2.

According to the characteristics of hydrological time series, 12 months is usually used as the observation period. The observed hydrological data such as water level and water potential, are formed into a sequence of feature vectors $\left[ x_1,x_2,\ldots ,x_n \right]$, where $x_i\in R^N$ and N is the number of features in the sequence data. Feature vector sequence $x=\left[ x_1,x_2,\ldots ,x_n \right]$is put after the first LSTM layer to obtain the new hidden layer vector $\left[ h_1^1,h_2^1,\ldots ,h_n^1 \right]$ and memory cell vector $\left[ c_1^1,c_2^1,\ldots ,c_n^1 \right]$ at moment *t*.

7 $$\begin{aligned} c_t^1,h_t^1=f_1 \left( h_{t-1}^1,x_t \right) \end{aligned}$$

The attention layer weights and sums the input feature vector sequence *x* and the hidden layer vector and memory cell vector output from the first LSTM layer to obtain the attention vector.

8 $$\begin{aligned} O'=\tanh {\left( {\varvec{W}}\left[ h_t^1,c_t^1 \right] +{\varvec{U}}x_i+{\varvec{b}}\right) } \end{aligned}$$

The attention weight $a_i$ for the importance of the *ith* feature at moment *t* is obtained by changing the sum of all the attention weights to 1 through the *softmax* function and represents the importance of the different features.

9 $$\begin{aligned} a_i=softmax \left( \omega ^TO' \right) \end{aligned}$$

where $\varvec{\omega }^T\in R^N$, $W \in R^{N \times \text{ Hidden } \text{ layer } \text{ size }}$, and   ${\varvec{U}}\in R^{N\times N}$ are weight matrices, and ${\varvec{b}}\in R^N$ is the item to be learned by the attention mechanism.

The attention layer assigns weights to the input feature vector sequence *x* to obtain a new vector ${\tilde{x}}=[\widetilde{x_1},\widetilde{x_2},\ldots ,\widetilde{x_n}]$.

10 $$\begin{aligned} {\tilde{x}}=\tanh (a*x) \end{aligned}$$

The new sequence of feature vectors ${{\widetilde{x}}}$ is used as input to the second layer of the LSTM network, and the information in the sequence data is extracted, memorised and learned. The mapping relation from ${\tilde{x}}_{t}$ to $h_t^2$ at time *t* can be obtained through the learning of the second layer of the LSTM network.

11 $$\begin{aligned} h_t^2=f_2(h_{t-1}^2,{\widetilde{x}}_t) \end{aligned}$$

The hidden layer vector output from the second layer of the LSTM network is dimensionally transformed in the fully connected layer. Finally, in the output layer, the length to be output is selected, and the result is transformed into the predicted dimensionality.

12 $$\begin{aligned}{}[y_1,y_2,\ldots ,y_m]=activation \left( {\textbf {H}}W_d \right) \end{aligned}$$

where $[y_1,y_2,\ldots ,y_m]$ denotes the prediction results in *m* time periods, *H* denotes the input matrix formed by $\left[ h_1^2,h_2^2,\ldots ,h_n^2 \right]$, $W_d$ denotes the weight matrix, and *activation* denotes the activation function.

## Whale optimisation algorithm

The whale optimisation algorithm (WOA) is a novel population intelligence optimisation algorithm that was proposed in 2016^20^. The algorithm simulates the bubble-net feeding method used in humpback whale hunting, and its performance far exceeds that of traditional algorithms. This algorithms consists of three main stages: surrounding prey, performing a bubble-net attack, and searching for prey.

## Surrounding prey

Humpback whales swim in the direction of the best positioned humpback whale when they locate and surround.It is assumed the best positioned humpback whale is the target prey.

13 $$\begin{aligned} \overrightarrow{D} = \left| \textbf{C}\cdot \overrightarrow{X^*}(t)-\overrightarrow{X}(t) \right| \end{aligned}$$

14 $$\begin{aligned} \overrightarrow{X}(t+1)=\overrightarrow{X^*}(t)-\overrightarrow{A}\cdot \overrightarrow{D} \end{aligned}$$

where *t* indicates the current number of iterations, $\overrightarrow{A}$ and $\overrightarrow{C}$ are vectors of coefficients, $\overrightarrow{{X^{*}}}$ is the currently obtained optimal humpback whale as a position vector, and $\overrightarrow{X}$ is the current humpback whale position vector. The optimal solution $\overrightarrow{{X^{*}}}$ will change with updates during the iterative process.

15 $$\begin{aligned} \overrightarrow{A}=2\overrightarrow{a}\cdot \overrightarrow{r}-\overrightarrow{a} \end{aligned}$$

16 $$\begin{aligned} \overrightarrow{C}=2\overrightarrow{r} \end{aligned}$$

$\overrightarrow{a}$ decreases linearly from 2 to 0 with the number of iterations.

17 $$\begin{aligned} \overrightarrow{a}=2-\frac{2t}{T_{max}} \end{aligned}$$

where *t* indicates the current number of iterations and $T_{max}$ is the maximum number of iterations.

## Performing a bubble-net attack

When hunting, whales blow bubbles to form bubble nets to chase their prey, and the following mathematical model is used to simulate this predatory behaviour.

Shrinkage envelope mechanism: The behaviour of whales feeding on their prey is simulated by decreasing the value of $\overrightarrow{a}$ during the iterative process. $\overrightarrow{A}$ also decreases as $\overrightarrow{a}$ decreases and fluctuates within the interval [− a,a]. When $\overrightarrow{A}$ is a random value in [− 1,1], then the new position of the humpback whale can be any position between the original position and the optimal individual position.

Spiral location update: The distance between the humpback whale at (*X*, *Y*) and the prey at $(X^*,Y^*)$ is first calculated, and then the spiral movement performed by the humpback whale is modelled using the spiral equation.

18 $$\begin{aligned} \overrightarrow{D'}=|\overrightarrow{X^*}(t)-\overrightarrow{X}(t)| \end{aligned}$$

19 $$\begin{aligned} \overrightarrow{X}(t+1)=\overrightarrow{D'}\cdot e^{bl}\cdot \cos {(2\pi l)}+\overrightarrow{X^*}(t) \end{aligned}$$

where (*X*, *Y*) is the position of the humpback whale, $(X^*,Y^*)$ is the position of the prey, $\overrightarrow{D^{\prime }}$ denotes the distance between the ith humpback whale and the target prey, *b* is a constant, and *l* is a random number between [-1,1].

As the humpback whale swims around its prey, it follows a spiral path. To model these two simultaneous behaviours, the same probability is used for updating of the whale’s position.

20 $$\begin{aligned} \overrightarrow{X}(t+1)=\left\{ \begin{array}{r}\overrightarrow{X^{*}}(t)-\overrightarrow{A} \cdot \overrightarrow{D}, p<0.5 \\ \overrightarrow{D^{\prime }} \cdot e^{b l} \cdot \cos (2 \pi l)+\overrightarrow{X^{*}}(t), p \ge 0.5\end{array}\right. \end{aligned}$$

where *p* denotes the probability between [0,1].

## Searching for prey

In the search phase, i.e, when $\overline{{\left| A\right| }}$ > 1, the humpback whale is not in the position of the best individual in the reference population. However, the position of a randomly selected humpback whale is updated with the aim of conducting a global search.

21 $$\begin{aligned} \overrightarrow{D}=\left| \overrightarrow{C}\cdot \overrightarrow{X}_{rand}-\overrightarrow{X}(t) \right| \end{aligned}$$

22 $$\begin{aligned} \overrightarrow{X}(t+1)=\overrightarrow{X}_{rand}-\overrightarrow{A}\cdot \overrightarrow{D} \end{aligned}$$

where $\overrightarrow{X}_{rand}$ is the position vector of a randomly selected humpback whale and $\overrightarrow{D}$ is the distance from the randomly selected humpback whale to the prey.

## Improved whale optimisation algorithm

The whale optimisation algorithm achieves good results in terms of the convergence accuracy and convergence speed and has the advantages of operation simplicity, and few parameters. However, this algorithm also has the problem of an imbalance between its global search ability and local exploitation ability, and it easily falls into local optimal solutions. A high-quality initialisation population contributes significantly to the performance of the algorithm in terms of the solution accuracy and convergence speed. To further improve the accuracy of the whale optimisation algorithm, an improved whale optimisation algorithm named the IWOA is proposed. The algorithm first uses a backwards learning approach to initialise the population followed by a nonlinear convergence factor for optimisation seeking at update time, achieving a balance between global and local search capabilities.

## Reverse learning to initialise populations

The quality of the initial population directly affects the subsequent iterations of the algorithm, and a high-quality population can effectively improve the convergence speed and accuracy of the iterative process. Due to the stochastic nature of intelligent optimisation algorithms, the initial populations of the original Whale Optimisation algorithm are generated in a random way, causing the WOA to be inefficient in its runtime search. To ensure the diversity of the initialised populations, a backwards learning approach is introduced into the WOA. N individuals from the initial population are combined with N individuals after reverse learning to form a new population with 2N individuals, and then the N individuals with the greatest diversity from the new population are selected by a ranking algorithm to form a new initialised population.

Reverse learning^21^ is based on specifying the range boundaries of variables and finding their corresponding reverse solutions via certain rules. If the size of the whale population is *N* and the search space is d-dimensional, the position of the ith whale in the d-dimensional space can be expressed as $X_i=\left( x_i^1,x_i^2,\ldots x_i^d \right) (i=1,2,3,\ldots N)$, $x_i^j\in \left[ a_i^j,b_i^j \right]$(j=1,2,3,...d), $a_i^j$ and $b_i^j$ denote the lower and upper bounds of $x_i^j$ respectively, $\widehat{x_l^J}$ denotes the new individual after reverse learning, and *rand* is a random number from 0 to 1.

23 $$\begin{aligned} \widehat{x_l^J}=\frac{a_i^j+b_i^j}{2}+rand* \left( x_i^j-\frac{a_i^j+b_i^j}{2}\right) \end{aligned}$$

where $x_i^j\in \left[ a_i^j,b_i^j \right] (j=1,2,...,d)$ denotes the coordinates of a single whale. The position of the ith whale in the d-dimensional space can be expressed as $X_i= \left( x_i^1,x_i^2,\ldots x_i^d \right) (i=1,2,3\ldots N)$, $a_i^j$ and $b_i^j$ denote the lower and upper bounds of $x_i^j$ respectively, $(a_i^j+b_i^j)/2$ denotes the average of the upper and lower bounds, *rand* is a random number from 0 to 1, and $rand*\left( x_i^j- \left( a_i^j+b_i^j \right) /2 \right)$ denotes the random part of the reverse learning. If rand is 1, then the position of the whale is unchanged. If rand is 0, the position of the whale is the midpoint of the upper and lower bounds. A new individual is created after each initial population individual is learned in reverse, and the number of individuals in the combined population is 2N.

The individuals in the population are stratified by a noninferiority sorting algorithm, which divides the individuals in the population into *L* levels according to the relationships between individuals. Individuals at the same level have the same rank, which is denoted as $Level_i$. The individuals in the first level are noted as $Level_1$, and the level rank is the highest. For individuals in the same stratum, the crowding distance sorting method is used.

24 $$\begin{aligned} Dis(i)=Dis(i)+\frac{f_M(i+1)-f_M(i-1)}{f_{Mmax}-f_{Mmin}} \end{aligned}$$

where *Dist*(*i*) denotes the crowding distance of an individual and the initial value is set to 0, i.e, $Dis(i) = 0$. $f_{Mmax}$ and $f_{Mmin}$ are the maximum and minimum values of the Mth objective function, respectively. $f_M (i+1)$ and $f_M (i-1)$ are the values of the Mth objective function for the two individuals on the same level as *i* and adjacent to it. The individuals of the population are ordered in the following way: each individual has two attributes, *Level*(*i*) and *Dist*(*i*). For any two individuals *i* and *j* in the population, *Level*(*i*) and *Level*(*j*) are compared when they are at different levels. If *Level*(*i*) < *Level*(*j*), then *i* is ordered higher than *j*; when they are at the same level, the crowding distance of the individuals is compared and the individual with the greater crowding distance is retained. That is, when $Dis(i)>Dis(j)$, *i* is ordered higher than *j*.

25 $$\begin{aligned} \textrm{i}\quad \text {better} \quad \textrm{j}=\left\{ \begin{array}{c}{\text {level}}(i)<{\text {level}}(j) \\ {\text {Dis}}(i)>{\text {Dis}}(j), \quad \text{ if } \quad {\text {Level}}_{i}= \text{ Level } _{j}\end{array}\right. \end{aligned}$$

## Convergence factor update

The traditional whale optimisation algorithm determines whether to perform a global or local search by means of the parameter $\overrightarrow{A}$. However the update of the parameter $\overrightarrow{A}$ relies mainly on the convergence factor $\overrightarrow{a}$ for linear changes. The use of linear transformations makes the convergence rate consistent, so a nonlinear convergence factor is designed in this paper.

26 $$\begin{aligned} \overrightarrow{a}=2-2sin \left( \mu \frac{t}{T_{max}}\pi +\varphi \right) \end{aligned}$$

where $T_{max}$ denotes the maximum number of iterations, $\mu$ and $\varphi$ are the relevant parameters. In this paper,$\mu =0.5$ and $\varphi =0$ are chosen in this paper.

## IWOA-optimised ALSTM model

**Figure 3:**
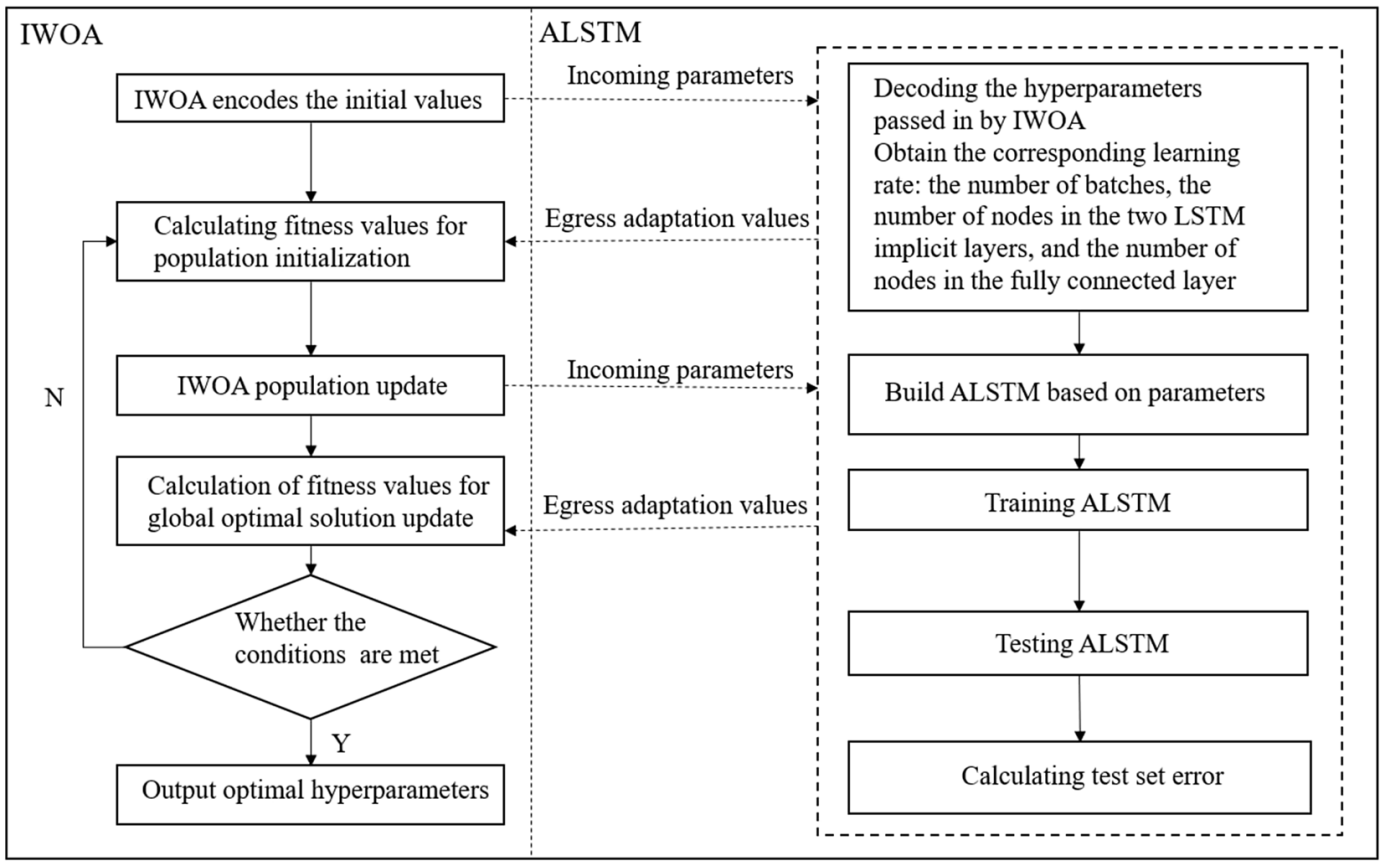
IWOA-optimised ALSTM structure.

The ALSTM model requires the determination of six main parameters, namely, the number of nodes in the first LSTM hidden layer, the number of nodes in the second LSTM hidden layer, the number of nodes in the fully connected layer, the learning rate, the number of batch processes and the number of iterations. With a large sample size, the prediction accuracy of a neural network model varies with the structure of the network. The learning rate determines the step size of the weight iterations; too large of a step size will result in a nonconverging model, and too small of a step size will result in slower convergence. A large batch size will reduce the training time and improve the stability of the model, but as the batch size increases, the performance of the model will decrease; as the number of iterations increases, the neural network will fit increasingly better and eventually enter overfit. In this paper, the IWOA algorithm is used to determine the above six parameters, and the optimised network model parameters are used as the final prediction model. The structure of the IWOA-optimised ALSTM is shown in Fig. 3.

The optimisation of the proposed ALSTM model using the IWOA proceeds in six main steps, which are explained below.

- Step 1: The maximum and minimum boundary values for the number of LSTM hidden layer neurons, the number of fully connected neurons, the learning rate, the number of batches, and the number of iterations are set, and the IWOA selects the minimum boundary value as the initial value and encodes it.
- Step 2: The IWOA is initialised with parameters such as the number of populations *N*, the maximum number of iterations $T_{max}$ and the probability *p*. The initial population selection is carried out by backwards learning and individual sorting of the populations, and the parameters are passed into the ALSTM model, which calculates the fitness value of the model and derives the current optimal solution $X^*$.
- Step 3: The IWOA population and the parameters $\overrightarrow{a}$ and $\overrightarrow{A}$. If $\therefore \overrightarrow{|A|}$ > 1, then a global merit search is performed; otherwise, a local merit search is performed and the population update is completed.
- Step 4: The updated information about the population parameters is passed into the ALSTM and the fitness value is calculated, overwriting the current optimal solution and its corresponding fitness value if it is smaller. If larger, then the current solution and its corresponding fitness are retained.
- Step 5: A determination on whether the training number has reached the maximum number of iterations $T_{max}$. If the training number reaches the maximum, the optimal ALSTM hyperparameters are obtained and assigned to the ALSTM model. If the number of iterations is less than $T_{max}$, return to step 3.
- Step 6: An ALSTM model is built based on the obtained optimal ALSTM hyperparameters to predict and analyse the hydrological time series.

## Experiment and analysis

To investigate the performance of the IWOA-ALSTM model, we verify the iterative convergence effect of the IWOA convergence factor and verify whether the IWOA can effectively perform a hyperparameter search for the ALSTM and predict the non-linear components of the water level time series. In this section, experiments are conducted on the nonlinear subcomponent series of hourly water level data from Hankou station in the Yangtze River Basin.

## Data and environment

**Figure 4:**
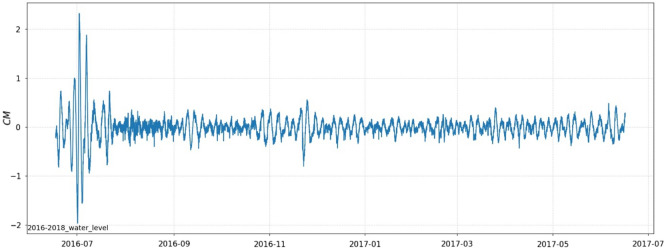
Water level nonlinear component behaviour.

The hydrological dataset for this experiment is derived from the nonlinear components of the measured hydrological data from Hankou Station in the Yangtze River Basin, with the raw data collected at a frequency of 60 minutes. The data were collected from 8:00 on June 17, 2016, to 8:00 on June 16, 2017, with a total of 8736 sets of experimental samples (including 106 sets of missing values). The first 7862 sets of data were selected as the sample data for training, and the final 874 sets of data were used as the test sample to test the accuracy of the model predictions. The nonlinear component variation curves of the water level at Hankou station are shown in Fig. 4.

## Data preprocessing

### Missing value filling

For missing values in the water level data, linear interpolation was used to fill in the values.

27 $$\begin{aligned} y=y_0+(x-x_0)\frac{y_1-y_0}{x_1-x_0} \end{aligned}$$

where $(x_0,y_0 )$ and $(x_1,y_1)$ are known coordinates. *x* is in the interval $[x_0,x_1]$.

#### Data normalisation

As obseved in the graphs of water level changes, the water levels fluctuate widely range and have different magnitudes. The data are processed using min-max normalisation and scaled to the [0,1] range.

28 $$\begin{aligned} X_{i}^{\prime }={\frac{X_{i}-X_{m i n}}{X_{m a x}-X_{m i n}}} \end{aligned}$$

where $X_{max}$ and $X_{min}$ are the maximum and minimum values of the sequence, respectively, $X_{i}^{\prime }$ denotes the normalised value, and $X_i$ denotes the element in the original sequence.

#### Data denormalisation

Normalising the data to between [0,1] facilitates the evaluation of the model’s performance and speeds up the convergence of the model, and data denormalisation is performed to retain data and observations on the same order of magnitude.

29 $$\begin{aligned} X_{pre}'=(X_{max}-X_{min})*X_{pre}+X_{min} \end{aligned}$$

where $X_{max}$ and $X_{min}$ are the maximum and minimum values, respectively, when normalisation is performed, $X'_{pre}$ is the predicted value after inverse normalisation and $X_{pre}$ is the original prediction.

#### Data disaggregation

The wavelet transform is a common data transformation technique that decomposes data. In this experiment, the nonlinear components of the measured hydrological data from Hankou Station in the Yangtze River Basin were used. To obtain the nonlinear components of the original data, wavelet decomposition techniques are used. The wavelet decomposition basis function uses the db wavelet, and the number of decomposition layers is chosen to be 6.

## Evaluation metrics

In this paper, the prediction model is evaluated using three evaluation metrics. In the following equation, *n* denotes the number of observations, $y_i$ denotes the actual hydrological data on day *i*, ${\widehat{y}}_l$ denotes the predicted hydrological data on day *i*, ${\bar{y}}$ denotes the actual sample mean, and $\bar{\hat{y}}$ denotes the predicted sample mean.

### Root mean square error

The root mean square error is used to evaluate the deviation of the predicted value from the actual value, and the range is $[0,+\infty )$, with a smaller value indicating a higher prediction accuracy.

30 $$\begin{aligned} \text {RMSE}=\sqrt{\frac{1}{n}\sum _{i=1}^n(y_i-\widehat{y_l})^2} \end{aligned}$$

#### Mean absolute error

The mean absolute error indicates the deviation between the predicted and actual values. When the fit is good, the MAE tends to zero, which indicates the prediction accuracy of the model.

31 $$\begin{aligned} \text {MAE}=\frac{1}{n}\sum _{i=1}^n|y_i-\widehat{y_l}| \end{aligned}$$

#### Nash efficiency factor

The Nash efficiency factor is used to assess the predictive capability of a hydrological model^22^. The closer to 1 the NSE is, the greater the confidence in the predictive effect of the model, and vice versa.

32 $$\begin{aligned} NSE=1-\frac{\sum _{i=1}^n(y_i-\widehat{y_l})^2}{\sum _{i=1}^n(y_i-{\bar{y}})^2} \end{aligned}$$

#### Scatter index

The scatter index is used to measure the ratio between the dispersion of predicted values and the dispersion of actual observations. When the SI is in the range of [0, +), the smaller the SI is, the closer the dispersion of the predicted values to the dispersion of the actual observations and the greater the accuracy of the predicted values.

33 $$\begin{aligned} \text {SI} = \frac{\sqrt{\sum _{i=1}^{n} \left( (\hat{y}_i - \bar{\hat{y}}) - (y_i - {\bar{y}})\right) ^2}}{n \cdot {\bar{y}}} \end{aligned}$$

#### Discrepancy ratio

The discrepancy ratio is an indicator used to assess the difference between predicted and observed values. The closer to 0 the DR value is the smaller the difference between the predicted and observed values; conversely, the larger the difference.^23^

34 $$\begin{aligned} \text {DR} = \frac{\sum _{i=1}^{n} \frac{\hat{y}_i}{y_i}}{n} - 1 \end{aligned}$$

## Performance analysis

### Comparative experiments on the effect of convergence factors

**Figure 5:**
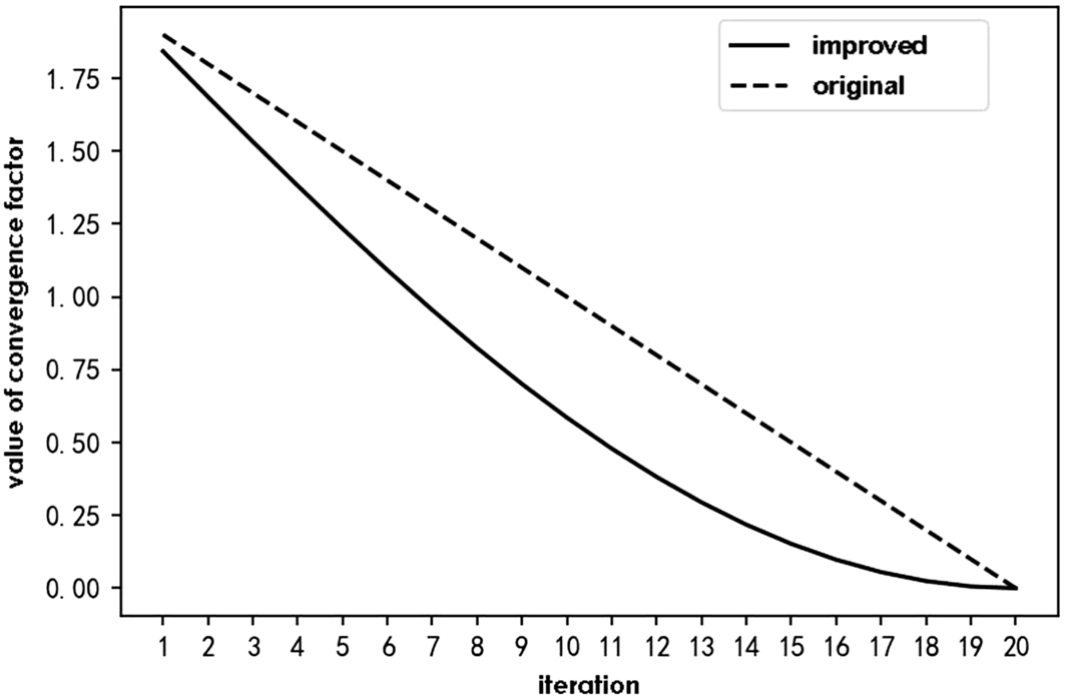
Convergence factor iteration diagram.

To verify the convergence effect of the nonlinear factor, the convergence effect of the IWOA convergence factor was first compared with that of the original WOA convergence factor. During the experiment, the iteration factors were iterated 20 times each to determine how their data changed. The change in the data can be determined by the change in the value of the image and the degree of slope of the lines. Figure 5 shows the convergence factor iteration diagram.

The maximum number of iterations $T_{max}$ is 20 in this figure. The solid line is the improved nonlinear convergence factor of the IWOA, and the dashed line is the linear convergence factor of the original WOA.

As observed from the graph, the improved convergence factor decreases as the number of iterations increased. During the iterative process, the first stage of descent is faster, and the second stage slows down. Thus, the convergence time of the algorithm decreases in the first stage, and the slowdown of the convergence rate in the second stage can improve the accuracy of the optimisation search.

#### IWOA optimisation effect comparison

**Figure 6:**
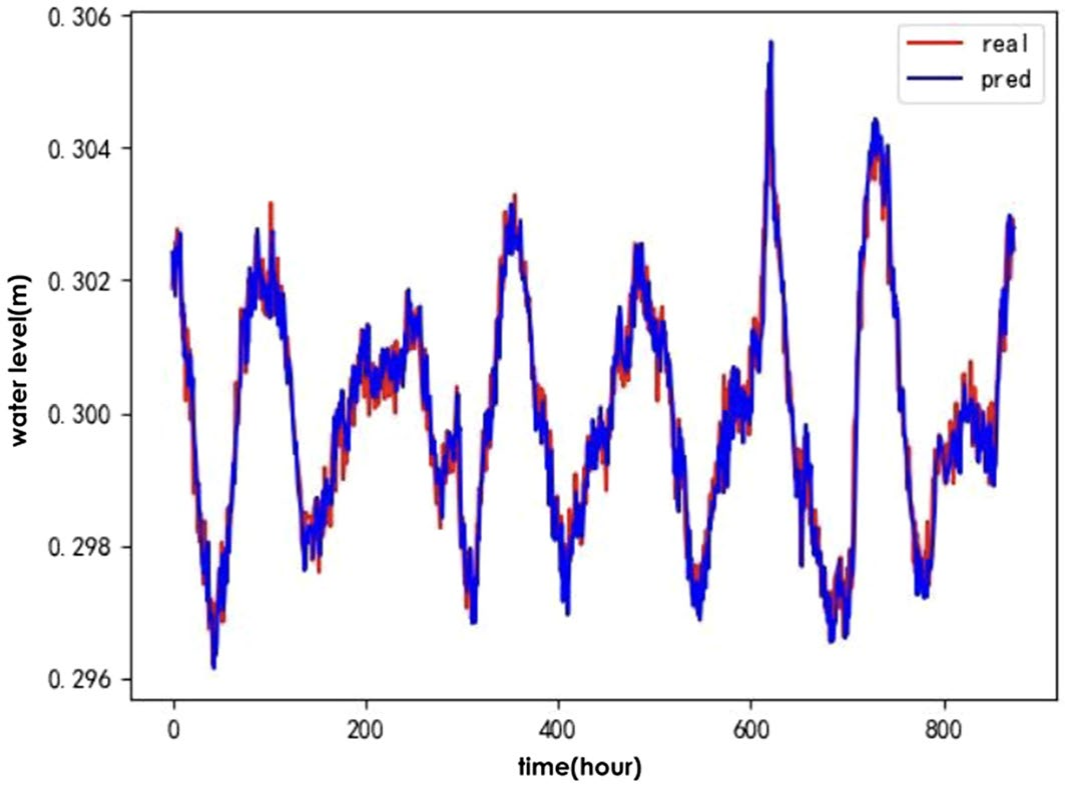
IWOA-ALSTM.

**Figure 7:**
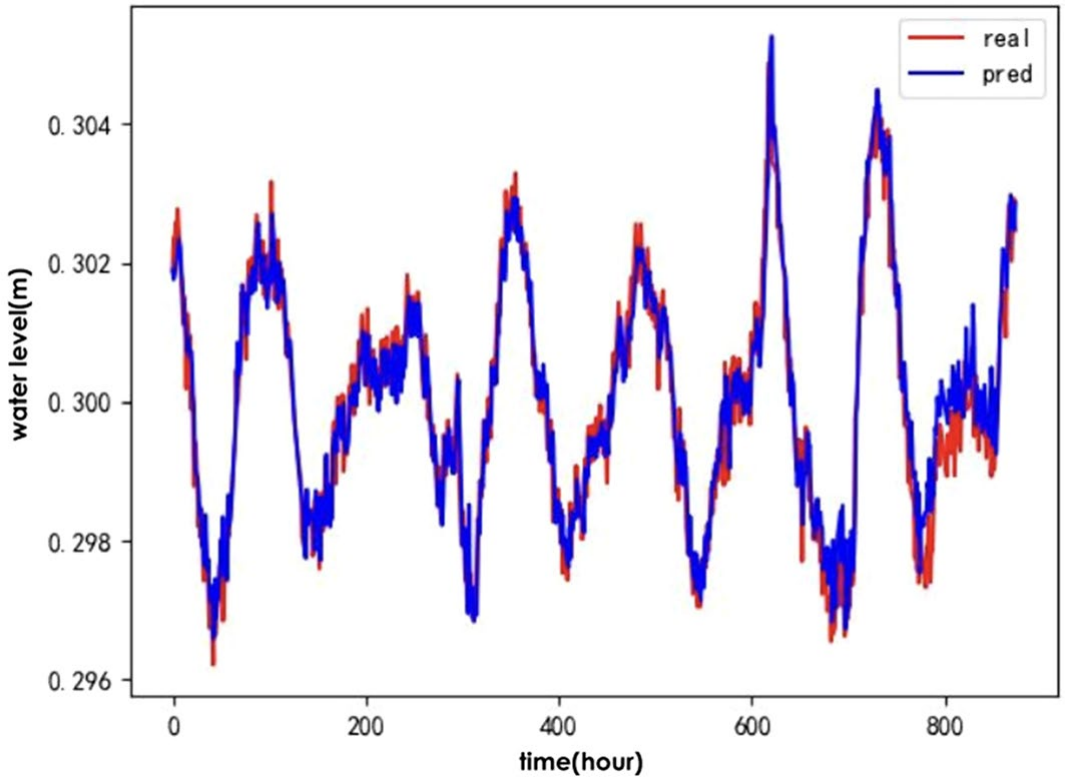
WOA-ALSTM.

**Figure 8:**
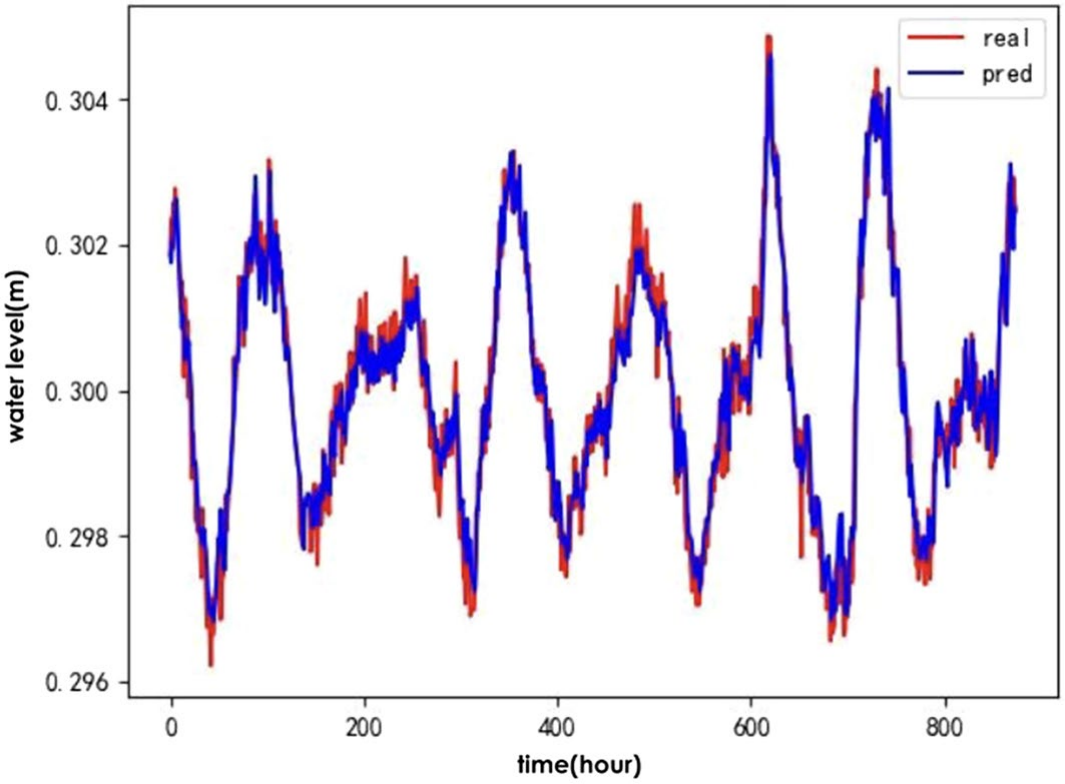
GA-ALSTM.

**Figure 9:**
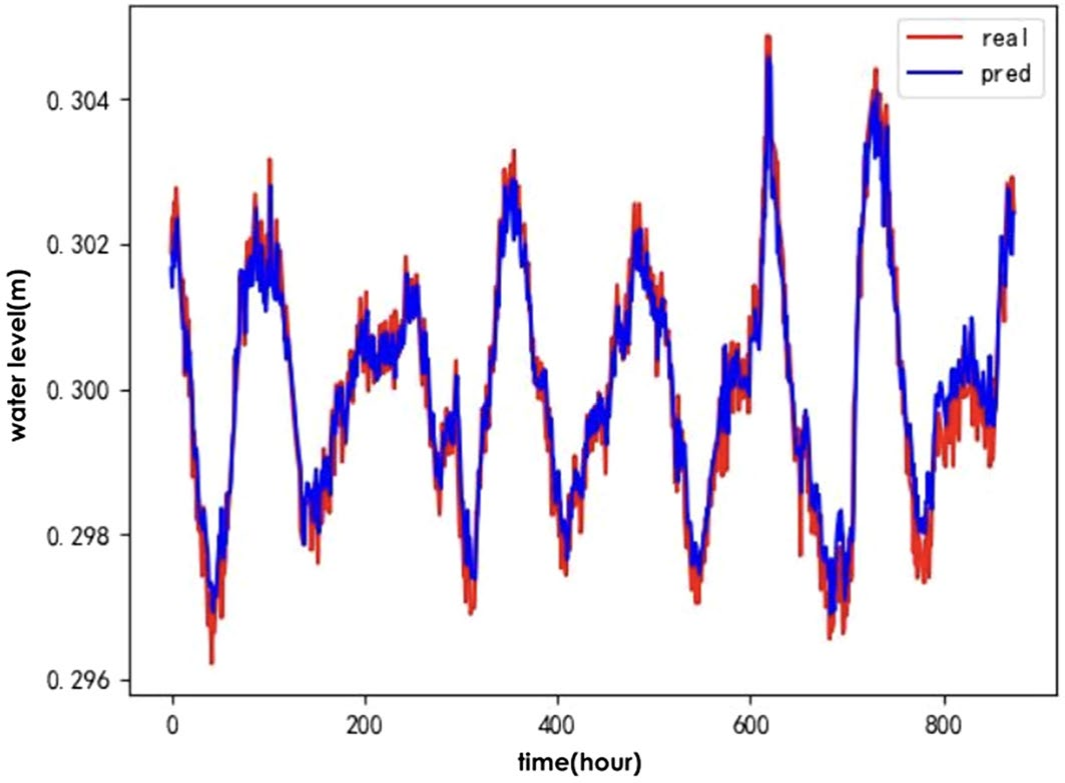
PSO-ALSTM.

To verify the effectiveness of the IWOA for optimising the ALSTM model, the following baseline algorithms were selected: the genetic algorithm(GA)^24^, Particle Swarm Optimisation (PSO)^25^ and the traditional whale optimisation algorithm (WOA). A comparison of the predicted and actual values for the test set of hydrological time series data after optimising the ALSTM model for each of the four algorithms is shown in Figs. 6, 7, 8, and 9.

The IWOA-ALSTM and WOA-ALSTM predictions are closer to the real values and can reflect the general trend of the real values. The GA-ALSTM and PSO-ALSTM prediction value curves, on the other hand, differ significantly from the actual value curves. Moreover, the IWOA-ALSTM prediction effect is better than the WOA-ALSTM prediction effect, as shown in the figure, from which it can be concluded that the hyperparameter search effect of the IWOA is better than that of the WOA.

The MAE, RMSE, NSE, SI, DR and model training time metrics are used to evaluate the performance of the hyperparametric optimisation model. Table 1 shows the error values for each model evaluation metric and the training times obtained after training for the four models.The RMSE, MAE, NSE, SI and DR of the IWOA-ALSTM model are 2.055, 0.063, 0.9248, 0.148 and − 0.003, respectively, and the RMSE, MAE, NSE, SI and DR of the WOA-ALSTM model are 2.923, 0.075, 0.9190, 0.159 and − 0.003, respectively. The RMSE, MAE, NSE, SI and DR are 13.225, 0.188, 0.9155, 0.249 and − 0.01 for the PSO-ALSTM model and 14.236, 0.915, 0.9135, 0.523, and 0.045 for the GA-ALSTM model respectively.

**Table 1:** Error values and training times for the four algorithms.

Evaluation indicators	GA-ALSTM	PSO-ALSTM	WOA-ALSTM	IWOA-ALSTM
RMSE	14.236	13.225	2.923	2.055
MAE	0.915	0.188	0.075	0.063
NSE	0.9135	0.9155	0.9190	0.9248
SI	0.523	0.249	0.159	0.143
DR	0.045	− 0.01	− 0.003	− 0.003
Time (s)	22.729	18.392	17.24	18.33

In terms of the prediction accuracy, the RMSE and MAPE of the IWOA-ALSTM model are 0.868 and 0.012 lower than those of the WOA-LSTM model, respectively. The NSE is 0.0058 greater and the SI of the IWOA-ALSTM model is better than that of the WOA-ALSTM model. The DR results of the IWOA-ALSTM model are the same as those of the WOA-ALSTM model but better than those of the PSO-ALSTM and GA-ALSTM models. The overall performance of these four models is ranked in increasing order, with GA-ALSTM yielding the worst results and IWOA-ALSTM achieving the best results. Moreover, PSO-ALSTM performs better than GA-ALSTM but worse than WOA-ALSTM. The GA and PSO algorithms are less effective at finding the best ALSTM model, and the WOA-ALSTM is significantly inferior to the IWOA-ALSTM. In terms of operational efficiency, the overall time overhead of the four models is the smallest for WOA-ALSTM, followed by IWOA-ALSTM. Although the training time of the IWOA-ALSTM model is slightly longer than that of the WOA-ALSTM model, it improves the prediction accuracy of the water level data. Thus, the experiments show that using the IWOA for ALSTM searches can result in better ALSTM hyperparameter values than can using the GA, PSO algorithm or WOA while improving the accuracy of hydrological time series predictions.

The above experiments show that the designed IWOA is slightly inferior to the WOA in terms of the identification time but is greatly improved in terms of the prediction accuracy. This result shows that the improved whale optimisation algorithm can effectively perform a parameter search for the designed ALSTM model and can improve the predictive power of the model.

## Conclusion

In this paper, an improved whale optimisation algorithm named the IWOA is proposed. This algorithm is used to perform a hyperparametric optimisation search for the ALSTM design, and experiments are carried out on nonlinear water level component data from Hankou station. Five different evaluation functions, i.e., the RMSE, MAE, NSE, SI and DR, are used to validate the accuracy of the proposed method. The experimental results show that the method proposed in this paper achieves the highest prediction accuracy. The IWOA-ALSTM model utilises an attention mechanism to extract more important features and the powerful parameter optimisation ability of the IWOA to achieve a more accurate prediction of hydrological time series.

Although this study presents promising results, there are some questions that deserve further consideration and exploration in the future:

1. This paper focuses on nonlinear water level data. However, in future investigations, we aim to encompass a broader spectrum of hydrological phenomena, including rainfall, evaporation, and water level measurements across diverse geographical locations and climatic conditions. To achieve this goal, we are committed to collaborating closely with hydrological research institutes to acquire a rich and comprehensive dataset.
2. In the future, we plan to conduct a thorough analysis of the strengths and weaknesses of the IWOA-ALSTM model across various prediction scenarios. This comprehensive assessment will guide us in refining and optimising the model to achieve enhanced performance. Our objective is to continually enhance the accuracy and versatility of model predictions, thereby advancing the field of hydrological prediction methods.
3. Future studies should also consider the impact of hydrological projections on water resource management, ecosystem conservation and communities. It is essential to understand and evaluate how these forecasts influence decision-making and policy in these interconnected domains.

## Data Availability

The data used to support the findings of this study are available from the corresponding author upon request.
